# Probing the Effect of the Non-Active-Site Mutation Y229W in New Delhi Metallo-β-lactamase-1 by Site-Directed Mutagenesis, Kinetic Studies, and Molecular Dynamics Simulations

**DOI:** 10.1371/journal.pone.0082080

**Published:** 2013-12-10

**Authors:** Jiao Chen, Hui Chen, Yun Shi, Feng Hu, Xingzhen Lao, Xiangdong Gao, Heng Zheng, Wenbing Yao

**Affiliations:** School of Life Science and Technology, China Pharmaceutical University, Nanjing, Jiangsu, China; Wake Forest University, United States of America

## Abstract

New Delhi metallo-β-lactmase-1 (NDM-1) has attracted extensive attention for its high catalytic activities of hydrolyzing almost all β-lactam antibiotics. NDM-1 shows relatively higher similarity to subclass B1 metallo-β-lactmases (MβLs), but its residue at position 229 is identical to that of B2/B3 MβLs, which is a Tyr instead of a B1-MβL-conserved Trp. To elucidate the possible role of Y229 in the bioactivity of NDM-1, we performed mutagenesis study and molecular dynamics (MD) simulations. Although residue Y229 is spatially distant from the active site and not contacting directly with the substrate or zinc ions, the Y229W mutant was found to have higher *k_cat_* and *K_m_* values than those of wild-type NDM-1, resulting in 1∼7 fold increases in *k_cat_*/*K_m_* values against tested antibiotics. In addition, our MD simulations illustrated the enhanced flexibility of Loop 2 upon Y229W mutation, which could increase the kinetics of both substrate entrance (kon) and product egress (koff). The enhanced flexibility of Loop 2 might allow the enzyme to adjust the geometry of its active site to accommodate substrates with different structures, broadening its substrate spectrum. This study indicated the possible role of the residue at position 229 in the evolution of NDM-1.

## Introduction

Metallo-β-lactamases (MβLs) caused antibiotic resistance has become a main therapeutic challenge in the clinic [1]. These enzymes are characterized by one or two zinc ions that are indispensable for enzyme activities [2]. The zinc ions in the active site activate a coordinated water molecule, forming a hydroxide moiety that acts as a nucleophile to attack the carbonyl group within the β-lactam ring [3]. MβLs have raised major concerns due to their broad hydrolytic spectrum on β-lactams and the absence of effective inhibitors for clinical treatment [4].

New Delhi metallo-β-lactamase (NDM-1) was first identified from *Klebsiella pneumonia*, isolated from a Swedish patient who had travelled to New Delhi [5]. NDM-1-positive strains can degrade almost all β-lactam antibiotics, including penicillins, cephalosporins, and the highly potent carbapenems [6]. NDM-1 genes are encoded on plasmids, facilitating their transmission across different bacterial strains. In addition, the widespread use of β-lactams has created selective pressure that promotes the dissemination of MβLs genes. Consequently, NDM-1 has spread to nearly every continent worldwide after its first identification and has become a formidable threat to human health [7].

Based on their amino acid sequences and zinc ion dependence, MβLs have been divided into subclasses B1, B2, and B3, all of which share a similar αβ/βα scaffold [8]. The subclass B1 enzymes have emerged as the most clinically significant MβLs with two zinc ions in the active site [9]. NDM-1 is grouped into subclass B1 and has been investigated by several structural studies [10]–[12]. The functional significance of several residues in the active site has been studied, including D124 (NDM-1 numbering is used throughout the paper, unless otherwise specified), which plays a critical role in the catalytic reaction with its exact function remains controversial [13]. Nevertheless, there have been relatively few studies on the role of non-active-site residues. The rapid evolution of NDM MβLs figured out that non-active-site residue mutations can also influence the activity of NDM-1. Two recent studies showed the increased hydrolytic activities of NDM-4 and NDM-5, which differ from NDM-1 by non-active-site amino acid substitutions (M154L for NDM-4, V88L and M154L for NDM-5), toward carbapenems and several cephalosporins [14], [15].

**Figure 1:**
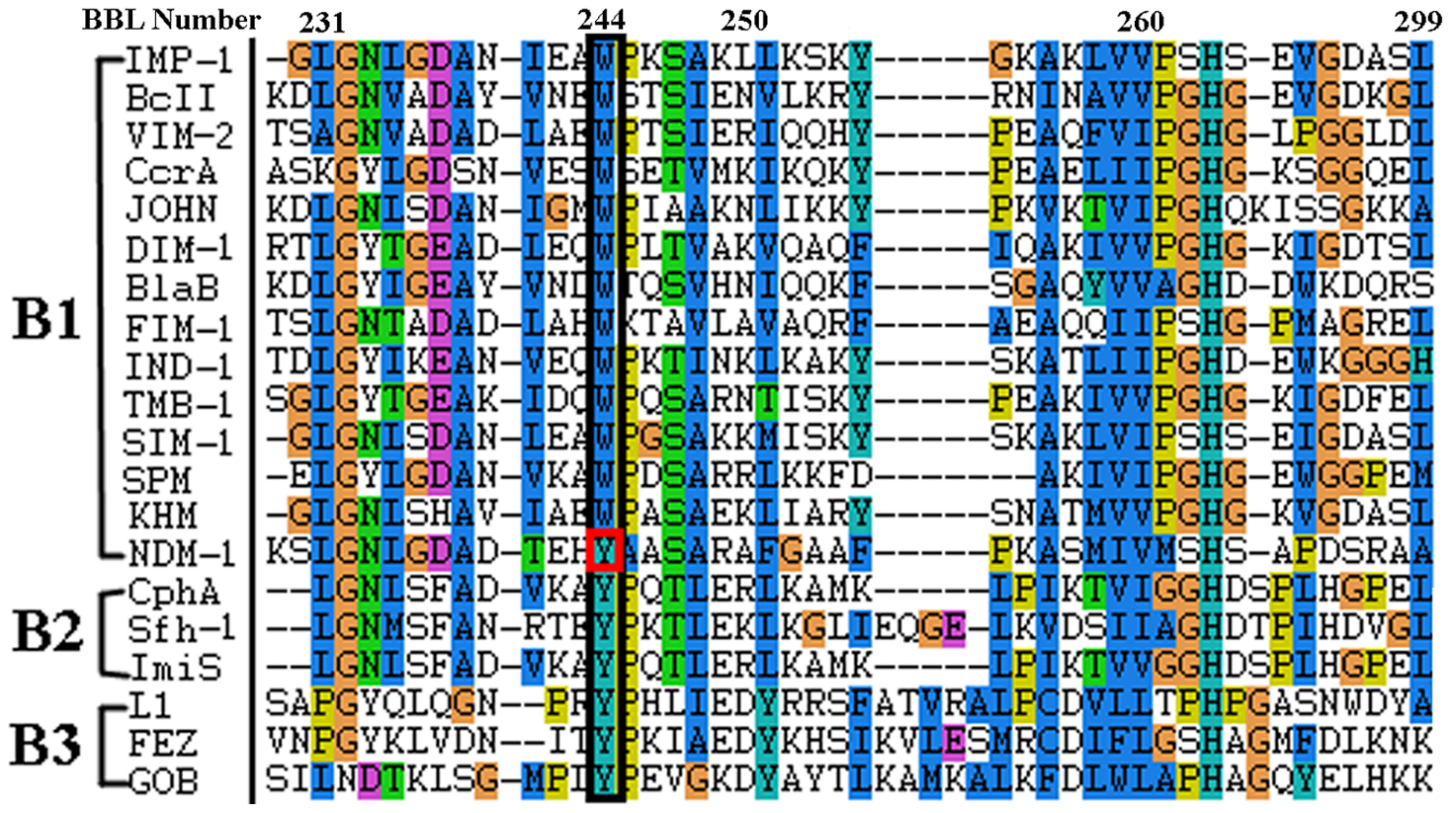
The sequence alignment of MβLs around position 244 (BBL number), parallel with position 229 in NDM-1.

Y229, which is structurally distant from the active site and not in direct contact with the substrate or zinc ions, has been shown to be an interesting residue in NDM-1 [12]. In subclass B1 MβLs, a Trp (W) residue is conserved at position 229 (position 244 in BBL numbering system [16], while in subclass B2 and B3 MβLs, it is a Tyr (Y) residue at this position (Fig. 1). Hence, NDM-1 is a remarkable exception in subclass B1, with a Tyr residue at position 229 that parallels subclass B2/B3 MβLs. It is reported that B2 (e.g. CphA, Sfh-1) and B3 (e.g. FEZ, L1) MβLs present narrower substrate spectrum than NDM-1 [6], [8], [17]. In order to probe the role of Y229 on the structure and activity of MβLs, we constructed the Y229W mutant of NDM-1 by site-directed mutagenesis and studied its kinetic properties. Molecular dynamics simulations have also been conducted to examine the structural changes upon mutation.

## Materials and Methods

### Site-directed mutagenesis and enzymatic activity assay

#### Construction of NDM-1 and Y229W mutant

The wild-type NDM-1 gene lacking the signal peptide was synthesized chemically and then cloned between EcoRI and Hind III restriction sites into the pET-28a plasmid harboring a kanamycin resistance gene. This *bla*_NDM-1_-encoding plasmid was used to introduce Y229W mutation by site-directed mutagenesis. The forward primer was 5′-GACACTGAGCACTGGGCCGCGTCAGC-3′ and the reverse primer was 5′-GCTGACGCGGCCCAGTGCTCAGTGTC-3′. The mutated gene was cloned between the same restriction sites into pET-28a as wild-type NDM-1.

The constructed plasmids encoding wild-type NDM-1 and Y229W mutant were transferred into *E.coli* BL21 (DE3). The enzymes were expressed and purified as previously described [18]. The N-terminal 6×His-Tag was removed by thrombin catalysis, followed by Ni column purification.

#### Determination of kinetic parameters

Hydrolysis of the antibiotics by wild-type NDM-1 and Y229W mutant was monitored by following the absorbance variation that resulted from the opening of the β-lactam ring [19]. All the measurements were performed at 30°C in the buffer of 50 mM HEPES, 250 mM NaCl, 200 µM ZnCl_2_, and pH = 7.25, using a UV-1800 spectrophotometer (Mapada, Shanghai, China). The molar extinction coefficients and wavelengths used in the assays were referred to those from [20], [21]. The steady-state kinetic parameters (*K_m_* and *k_cat_*) were determined at least three times by fitting the concentration dependence of initial rate measurements to the Michaelis-Menten equation.

#### Determination of minimum inhibitory concentrations (MICs)

MICs for *E.coli* BL21 (DE3) harboring NDM-1 and Y229W mutant were determined by a two-fold serial dilution method on 96-well microplates. *E.coli* BL21 (DE3)/pET-28a was used as negative control. The bacteria were diluted to 1×10^6^ CFU/ml by Luria-Bertani (LB) broth containing 50 µg/ml kanamycin and then incubated at 37°C for 12 hours after adding different concentrations of antibiotics. A reading at 600 nm was taken to determine the MICs.

### Molecular dynamics analysis of NDM-1 and Y229W mutant

#### Initial model building

Molecular dynamics (MD) simulations were employed to investigate the dynamic properties of NDM-1 and the Y229W mutant with/without the hydrolyzed Ampicillin (PDB entry: 3Q6X). The structure of Y229W mutant was built in Discovery Studio 2.5.5. Prior to modeling, all crystal waters were removed except for the bridging water, which was assumed to exist in hydroxide form between the two zinc ions. The bridging water was thus parameterized as a hydroxide ion in the MD simulations of the apo-enzyme (without substrate) and was deleted in enzyme-substrate systems.

Zinc ions were modeled as “cationic dummy atoms” (CaDA), which allowed for modeling of the binuclear Zn-containing proteins without having to covalently attach the coordinating residues to the Zn atoms. In this approach, the zinc's vacant 4s4p^3^ orbitals that accepted electron density of the four ligands were modeled by four identical dummy atoms, which were covalently bonded to the zinc nucleus and carried a partial charge of +0.5e each [22]. These dummy atoms have been shown to keep the ligands in the correct orientation, which enables a stable simulation of the tetrahedral coordination of the zinc atoms in IMP metallo-β-lactamases [23], [24].

In our model, zinc-coordinating histidines 120, 122, 189 and 250 were treated as histidinates, while Cys208 was assumed to be deprotonated at the S_γ_ position. Atom charges of the substrate were calculated using the RESP method [25] encoded in the AMBER 12.0 software package [26] at the HF/6-31G* level. As a comparison, the wild-type NDM-1 (PDB entry 3Q6X) was modeled with the same method as the Y229W mutant. Zinc dummy atoms were created using zinc, histidinate and hydroxide force field parameters provided by [22].

#### Molecular dynamics

Molecular dynamics simulations were performed with AMBER12.0. Each model was solvated in a periodic box of TIP3P [27] water molecules that extend 10 Å from the protein atoms. Counter-ions were added to neutralize the simulation systems. The ff12SB force field was used for the protein systems. To remove possible poor contacts between the solute and solvent, energy minimization (1000 steps for the water molecules followed by 3000 steps for the whole system) were performed before MD simulations. The SANDER program included in AMBER 12.0 was used to conduct the MD simulations at constant temperature and pressure with a time step of 1 fs. The temperature (300 K) and pressure (1.0 atm) of the system was controlled by the Langevin algorithm [28]. Electrostatic interactions were calculated using the particle-mesh Ewald method [29], and the non-bonded cutoff was set to 10.0 Å. The SHAKE algorithm [30] was used to constrain bonds involving hydrogen atoms. Each system was equilibrated for 500 ps under a NPT ensemble at a constant temperature of 300 K. Following this equilibration, production MD simulations were conducted for 20 ns for all systems with a time step of 1 fs.

#### Analysis of simulations

The ptraj analysis program within AMBER 12.0 was used for the calculations of the root mean square deviation (RMSD), the root mean square fluctuation (RMSF) and the existence of hydrogen bonds.

Based on the equilibrated dynamics trajectory (the last 4ns), the binding free energy of each complex system was calculated using the MMPBSA.py module. A total of 400 snapshots from the trajectory were extracted every 20 ps, and the MM/PBSA and MM/GBSA calculations were performed on each snapshot. The binding free energies (ΔG) were computed according to the following equations [31]:
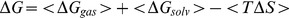
(1)

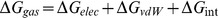
(2)

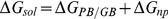
(3)

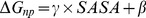
(4)where <…> indicates an average of an energy term along the MD simulation trajectory. ΔG_gas_ and ΔG_solv_ represent the vacuum and solvation binding free energies, respectively. –TΔS is the entropic contribution, which is not considered in the relative free energy analysis. ΔG_gas_ includes an intermolecular electrostatic term (ΔG_elec_), a van der Waals term (ΔG_vdW_), and an internal energy term (ΔG_int_). ΔG_solv_ is divided into the electrostatic solvation energy (ΔG_PB/GB_) and the nonpolar solvation energy (ΔG_np_). γ is the surface tension proportionality constant and β is the offset value. The solvent accessible surface area (SASA) was estimated by the MSMS algorithm with a probe radius of 1.4 Å [32].

## Results

### Experimental results

#### Kinetic parameters

Steady state kinetic parameters of wild-type NDM-1 and Y229W mutant with different antibiotics are summarized in Table 1. The *K_m_* values of Y229W mutant increased 1.5–5 times compared with wild-type NDM-1, while the catalytic constant *k_cat_* showed greater increases of 12–25 times against Penicillin G, Cefuroxime, Ceftizoxime and Meropenem. Thus, their increases in the hydrolytic efficiency (3∼7 folds) upon Y229W mutation was mainly due to the increase in the turnover rate rather than binding affinities. Specifically, the *k_cat_* of Penicillin G showed the largest increase after Y229W mutation, from 34±1.5 s^−1^ to 849±28 s^−1^, resulting in a 6 fold increase in *k_cat_/K_m_*. For Ampicillin, the Y229W mutant showed similar *K_m_* and *k_cat_* values to those of wild-type NDM-1, and no significant change was observed in *k_cat_/K_m_*.

**Table 1:** Kinetic parameters of the wild-type NDM-1 and the Y229W mutant.

Antibiotics	Wild-type NDM-1			Y229W mutant		
	*K_m_*	*k_cat_*	*k_cat_/K_m_*	*K_m_*	*k_cat_*	*k_cat_/K_m_*
	(µM)	(s^−1^)	(s^−1^/µM)	(µM)	(s^−1^)	(s^−1^/µM)
Penicillin G	178±55	34±1.5	0.19	737±52	849±28	1.15
Ampicillin	193±63	139±5	0.72	297±43	253±13	0.85
Cefuroxime	17±2	6.5±0.3	0.38	27±3	75±6	2.78
Ceftizoxime	21±5	3.9±0.3	0.18	101±25	53±3	0.52
Meropenem	59±2	35±2	0.59	268±15	435±82	1.62

#### Antibiotic susceptibility tests

To determine the antibiotic susceptibility of Y229W mutant and wild-type NDM-1, the MICs of Penicillin G, Ampicillin, Cefuroxime, Ceftizoxime and Meropenem were measured (Table 2). The *E.coli* BL21 harboring Y229W mutant showed strong resistance to Penicillin G, yielding a MIC of 256 µg/ml. The MICs of Ampicillin, Cefuroxime, and Meropenem decreased at least two folds after mutation, whereas a 8 fold decrease was observed in the case of Ceftizoxime. Therefore, the Y229W mutant rendered the host strain more susceptible to all tested antibiotics.

**Table 2:** MICs (µg/ml) for *E.coli* BL21 (DE3) host strain harboring the wild-type NDM-1 and Y229W enzymes.

Enzymes harbored in *E.coli* BL21 (DE3)	Antibiotics (µg/ml)					
	Penicillin G	Ampicillin	Cefuroxime	Ceftizoxime	Meropenem	
Wild-type NDM-1	>256	>256	256	64	16	
Y229W mutant	256	128	128	8	8	

### Molecular dynamics

#### Overview of the structure around Y229

**Figure 2:**
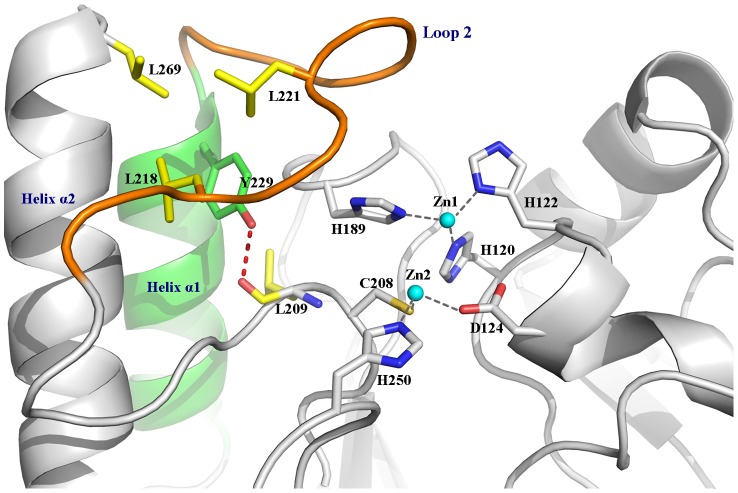
The interactions around Y229 and the active site residues of NDM-1.

The active site of NDM-1 is located at the bottom of a shallow groove enclosed by two important loops, namely Loop 1 and Loop 2. The role of Loop 1 and the N-terminal of Loop 2 has been investigated by both experimental and computational methods [33], [34]. The flexibility presumably assists in the structural rearrangement of the active site during catalytic turnover, granting interactions with a variety of substrates in B1 metallo-β-lactamases. Residing at the N-terminal of the helix α1, Y229 may play an important role in controlling the stability and flexibility of Loop 2, as it can form extensive hydrophobic interactions with neighboring residues, including L209, L218, and L221 in Loop 2, and L269 in helix α2 (Fig. 2). In addition, a hydrogen bond is also formed between the phenolic hydroxyl of Y229 and the backbone oxygen of residue L209. To investigate the role of the residue at 229 in the evolution of enzymatic activity, Y229 was selected to be mutated to Trp.

#### Dynamics and flexibility of the enzymes

**Figure 3:**
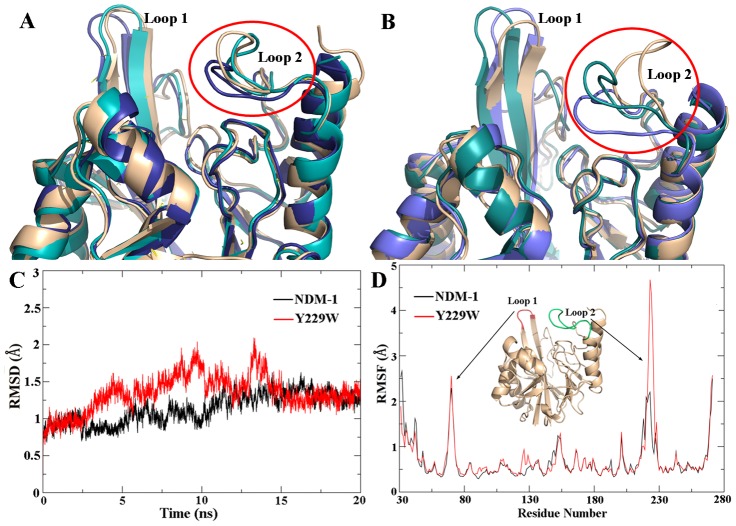
Distinct conformations of Loop 2 obtained by clustering the MD trajectory of the enzymes. (A) Superimposition of wild-type NDM-1; (B) Superimposition of Y229W mutant. (C) and (D) are the time evolution of the RMSDs and RMSFs of NDM-1 (black curve) and Y229W mutant (red curve) measured against the corresponding starting structures.

The root-mean-square deviation (RMSD) values of wild-type NDM-1 and Y229W mutant against the starting structures were calculated. The RMSDs of backbone atoms stabilized after 11 ns of simulation for NDM-1 and 15 ns for the Y229W mutant system, indicating that the Y229W mutant underwent a larger conformational change to reach equilibrium (Fig. 3C). The stabilized RMSD values of the final 4 ns insured the reliability and suitability of these MD trajectories for further analysis.

The root-mean-square fluctuation (RMSF) of Ca atoms was also calculated with respect to the starting structures (Fig. 3D). In the enzyme systems, Loop 2 was significantly more flexible in Y229W mutant than in NDM-1. As expected, Loop 1 was also found to be fluctuant in both systems, although the fluctuation was less different between wild-type NDM-1 and Y229W mutant.

Distinct conformations of Loop 2 were obtained by clustering the MD trajectories of wild-type NDM-1 and Y229W mutant. The superimpositions of the conformations were shown in Fig. 3A and Fig. 3B. For both systems, the conformations superimposed well except in the flexible regions of Loop 1 and Loop 2. To facilitate substrate hydrolysis, the active site of the enzyme needs both stability and flexibility. Y229W mutation enhanced the flexibility of Loop 2, which could increase the kinetics of both substrate entrance (kon) and product egress (koff). This might be caused by the different steric and electrostatic characteristics between Trp and Tyr.

#### Hydrogen bond analyses of Loop 2

In order to investigate the reason of increased flexibility of Loop 2 after mutation, hydrogen bond analyses were performed for the last 4 ns of MD simulations. Hydrogen bonds were defined by a donor-acceptor distance of smaller than 3.5 Å and a donor-hydrogen-acceptor angle of larger than 120°. Overall, 13 hydrogen bonds existed in the Loop 2 (residues from 214 to 229) of wild-type NDM-1 and 10 hydrogen bonds existed in Y229W mutant (Table 3). The hydrogen bonds accepted by N220 in NDM-1 were not observed in Y229W mutant. In wild-type NDM-1, the occupancies of hydrogen bonds involving D225 were all above 99%, much larger than those in Y229W mutant. Such weaker hydrogen bonding interactions could contribute to the increased flexibility of Loop 2 in Y229W mutant. Moreover, T226 on Loop 2 of Y229W mutant formed hydrogen bonds with A230 on helix α1 and L269 on helix α2, facilitating the movement of Loop 2 away from Loop1, which resulted in an increased volume of the active site compared with that of wild-type NDM-1.

**Table 3:** Hydrogen bonds existing in Loop 2 (residues from 214 to 229) of wild-type NDM-1 and Y229W mutant, and their occupancies during the last 4 ns of MD simulations.

NDM-1			Y229W mutant		
Donor	Acceptor	Occupancy (%)	Donor	Acceptor	Occupancy (%)
K214@ N	D212@OD1	84.14	K214@ N	D212@OD1	88.96
H261@ NE2	A215@O	90.19	H261@ NE2	A215@O	98.06
A215@ N	D212@O	99.55	A215@ N	D212@O	98.00
A224@ N	N220@O	99.76	——	——	——
D223@ N	N220@O	98.04	——	——	——
G222@ N	N220@OD1	85.64	——	——	——
——	——	——	W229@ N	D225@O	55.38
——	——	——	H228@ N	D225@O	53.72
E227@ N	D225@OD1	99.10	——	——	——
H228@ N	D225@OD1	99.43	——	——	——
D225@ N	G188@O	99.45	——	——	——
——	——	——	A230@ N	T226@O	85.96
——	——	——	T226@ OG1	L269@O	57.93
S232@ N	H228@O	96.63	S232@ N	H228@O	98.58
A231@ N	H228@O	60.30	——	——	——
Y229@ OH	L209@O	99.83	W229@ NE1	L209@O	98.75
A233@ N	Y229@O	93.83	A233@ N	W229@O	96.86

Only H-bonds with occupancies >50% are shown.

—— Not observed.

#### Interactions of NDM-1 and Y229W mutant with the hydrolyzed Ampicillin

**Figure 4:**
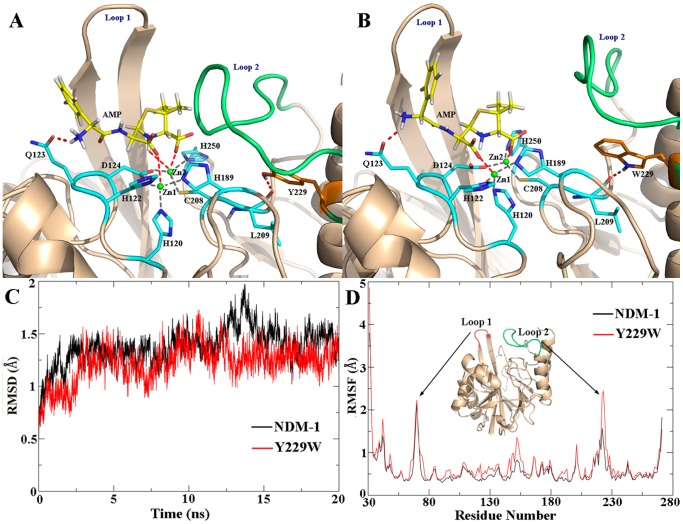
The central structure of the major cluster in the last 4 ns of (A) wild-type NDM-1 complexed with hydrolyzed Ampicillin, (B) Y229W mutant complexed with hydrolyzed Ampicillin. The time evolution of (C) RMSDs and (D) RMSFs of wild-type NDM-1 (black curve) and Y229W mutant (red curve), measured against the corresponding starting structures.

To investigate the mutagenesis effect on the plasticity of protein and ligand binding, MD simulations of the enzyme-hydrolyzed Ampicillin complexes were performed. Both the wild-type NDM-1 and Y229W mutant systems reached equilibrated states after 16 ns. Compared with the apo-enzyme systems, the RMSDs of enzyme-product complexes were more stable for both wild-type NDM-1 and Y229W mutant (Fig. 4C). Although Loop 2 in Y229W mutant complex was still more fluctuant than that in wild-type NDM-1 complex, the RMSF values were much smaller for both systems (Fig. 4D), indicating the loop could be stabilized by interactions with the substrate molecules.

The last 4 ns of each MD trajectory was clustered based on heavy-atom RMSDs, and the central structure of the major cluster (the member with the smallest average RMSD values to all other members of the major cluster) was extracted to serve as the representative structure of each MD simulation. The binding conformations of the representative structures (Fig. 4A and 4B) showed few changes in the active site in comparison with the crystal structure. Zn1 was coordinated by three histidine residues (H120, H122, and H189) and Zn2 was coordinated by D124, C208, and H250. The carboxylate group of the β-lactam ring coordinated to Zn2 through one oxygen atom and the newly formed carboxylate group after hydrolysis coordinated to Zn1. The nitrogen near the phenyl ring formed hydrogen bond with OE1 of Q123. The nitrogen atom of the β-lactam ring coordinated to Zn2 in the NDM-1 complex. However, this interaction was not present in the Y229W mutant complex. The hydrolyzed Ampicillin also formed hydrophobic interactions with Loop 1 and Loop 2, stabilizing both mobile loops.

**Figure 5:**
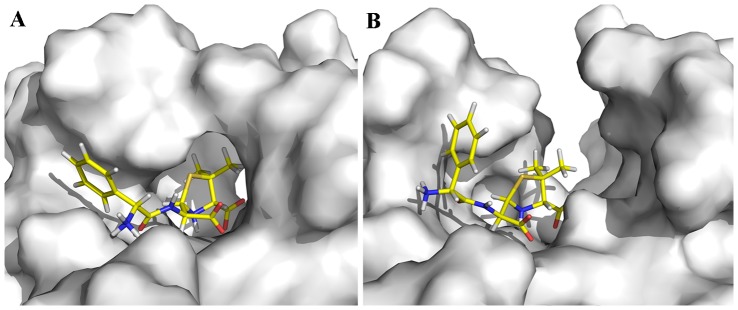
Surface representation of the central structure in the major cluster of the last 4 ns, showing the binding groove for substrates in (A) wild-type NDM-1, (B) Y229W mutant.

Remarkably, the active-site cleft under Loop 1 and Loop 2 was broader in Y229W mutant (Fig. 5B) than that in wild-type NDM-1 (Fig. 5A). The enlarged binding groove of Y229W mutant can easily accommodate β-lactam antibiotics with different R groups. Besides, the hydrolyzed product could also exit from the binding groove faster thanks to less steric hindrance.

#### Binding free energy calculation

The binding free energy (MM/PBSA and MM/GBSA) of wild-type NDM-1/Y229W mutant with the hydrolyzed Ampicillin was calculated (Table 4). Both the MM/PBSA and MM/GBSA values suggested the hydrolyzed Ampicillin bound tighter to wild-type NDM-1 than to Y229W mutant, leading to an easier egress for the product of Y229W mutant. This correlates with the larger experimental *k_cat_* values of Y229W mutant. These calculations also imply that the binding free energy of the original substrate with wild-type NDM-1 is greater than Y229W mutant as well, which is in conformity with our experimental *K_m_* values. Thus, we propose that the product exiting and substrate entering might be the rate-limiting step in wild-type NDM-1- and Y229W-mediated β-lactams hydrolysis.

**Table 4:** The binding free energies (hydrolyzed Ampicillin as the substrate) calculated by the MM-PBSA and MM-GBSA method.

Enzyme	Binding free energy model	ΔG_gas_	ΔG_solv_	ΔG
		(kcal/mol)	(kcal/mol)	(kcal/mol)
NDM-1	MM/PBSA	−124.82	96.77	−28.05
	MM/GBSA	−124.82	75.29	−49.53
Y229W	MM/PBSA	−72.29	57.51	−14.78
	MM/GBSA	−72.29	52.80	−19.49

## Discussion

NDM MβLs have gained a lot of attention owing to their broad hydrolytic spectrum on β-lactams and the current lack of effective β-lactamase inhibitors against NDM-carrying superbugs. In addition, continuous evolution of drug-resistant bacteria leads to the emergence of new NDM variants, which makes the situation more discouraging. To date, 9 NDM variants have been identified or assigned (http://www.lahey.org/Studies/other.asp#table1, data collected before June 25, 2013). Some variants obtained increased enzymatic activities by mutating only one or two remote amino acids, suggesting important roles of these non-active-site residues in the function of NDM MβLs. Numerous studies have been carried out to explore the substrate recognition and catalytic mechanism, and most of them focused on active-site residues such as D124, K211 and N220 [13], [35].

Position 229 (244 in BBL numbering system) is very interesting since it may offer some evolutional clues of MβLs development. In most of the subclass B1 MβLs, a highly conserved Trp is observed, whereas in subclass B2/B3 MβLs, it is a Tyr. As we all know, most subclass B2/B3 MβLs exhibit narrower substrate spectrums than subclass B1 MβLs [2]. Unexpectedly, the broad-spectrum NDM-1 (subclass B1) employs a residue Tyr at position 229, which is usually conserved in B2/B3 MβLs. This prompted us to question whether the non-active-site substitution Y229W can influence the activity of NDM-1. Yu Guo *et al.* suggested that the peripheral residue Tyr229 may not directly participate in the enzymatic action of NDM-1, as confirmed by the minor effect of the Y229W mutation, but the conservation of this residue and its functional role in catalytic reaction in MBL superfamily should need further investigation [36]. Herein, we constructed the Y229W mutant of NDM-1 by site-directed mutagenesis and applied molecular dynamics simulations in order to investigate its effect on the structure and activity of NDM-1.

Biochemical analyses showed a simultaneous increase of *K_m_* and *k_cat_* upon Y229W mutation. For most of the tested antibiotics, the *k_cat_* values exhibited larger increase than *K_m_*, and thereby increased the catalytic efficiency (*k_cat_*/*K_m_*) by 1∼7 folds. However, *E coli.* carrying Y229W mutant showed reduced resistance to the tested antibiotics, which were inconsistent with the increased *k_cat_*/*K_m_* values. This might be caused by the decreased efficiency of mutant expression and culture conditions.

The non-active-site mutation Y229W may affect the catalytic efficiency via three different manners: 1) changing the kinetics of substrate entry or product exit by reducing or enlarging the volume of active-site pocket; 2) affecting the substrate binding by residue-residue interaction; 3) altering the stability of the three-dimensional (3D) structure [37]. To clarify this, MD simulations were performed to analyze the mutagenesis effect on Y229W mutant.

The substrate-enzyme interaction analysis (Fig. 4) revealed that the mutagenesis does not alter the catalytic properties of the enzyme. Superimposition of the conformations showed that Loop 2 is remarkably more flexible in Y229W mutant than that in wild-type NDM-1 (Fig. 3). The RMSF values also supported this point. The flexibility of Loop 2 in Y229W mutant might be caused by weaker interactions around the residues on Loop 2. For example, the number of hydrogen bonds around Loop 2 in Y229W mutant is less and some of the hydrogen bond occupancies are lower. In addition, the hydrophobic interactions between W229 and L221, L269 disappeared as their side chains deviated from the original direction.

Through strict computational means, the study of Freddie R. Salsbury Jr. *et al.* suggested that the flip of a loop indirectly involved in substrate binding can affect the catalytic efficiency and/or substrate specificity of an MBL while retaining a di-zinc active site [38]. Our study is in agreement with this point. We propose that the flexible Loop 2 could affect substrate entry and product exit by enlarging the active-site pocket as shown in the surface representation of substrate-enzyme complexes (Fig. 5). The broader active-site cleft under Loop 1 and Loop 2 in Y229W mutant can easily accommodate β-lactam antibiotics with different R groups and accelerate the egress of the hydrolyzed product, possibly contributing to increased *k_cat_* values in Y229W mutant. Meanwhile, the larger active-site pocket could also decrease the binding energies (MM/PBSA and MM/GBSA) of the substrate with Y229W mutant, corresponding to the increased *K_m_* values. Therefore, we propose that substrate-entry/product-release might be the rate-limiting step in a catalytic cycle.

## Conclusion

This study highlighted the importance of the non-active-site residue at position 229 in the evolution of NDM MβLs. The mutation from Tyr to Trp may not influence the overall structure of NDM-1, but it was shown herein to increase the flexibility of Loop 2 and broaden the binding pocket, which endowed the enzyme with increased turnover rates and decreased binding affinities. It is also implied that the Y229W mutant has higher structural flexibility near the active site than that of wild-type NDM-1, which probably broadens the substrate spectrum of the enzyme.
